# Whole-genome resequencing to investigate the genetic diversity and the molecular basis underlying key economic traits in indigenous sheep breeds adapted to hypoxic environments

**DOI:** 10.3389/fvets.2025.1605252

**Published:** 2025-07-02

**Authors:** Dehong Tian, Buying Han, Xue Li, Quanbang Pei, Baicheng Zhou, Kai Zhao

**Affiliations:** ^1^Key Laboratory of Adaptation and Evolution of Plateau Biota, Qinghai Provincial Key Laboratory of Animal Ecological Genomics, Northwest Institute of Plateau Biology, Chinese Academy of Sciences, Xining, China; ^2^College of Life Science, University of Chinese Academy of Sciences, Beijing, China; ^3^Key Laboratory of Adaptation and Evolution of Plateau Biota, Northwest Institute of Plateau Biology, Chinese Academy of Sciences, Xining, China; ^4^Qinghai Sheep Breeding and Promotion Service Center, Gangcha, China; ^5^Qinghai Yak Breeding and Promotion Service Center, Datong, China

**Keywords:** indigenous breeds, GWAS, hypoxic adaptability, production, transcriptome

## Abstract

Under the combined effects of long-term natural selection and artificial domestication, Tibetan sheep on the Qinghai-Tibet Plateau have evolved distinct ecotypes to survive extreme high-altitude conditions, including hypoxia, cold, and low oxygen levels. These ecotypic variations not only serve as an ideal model for studying plateau livestock adaptation but also harbor valuable genetic diversity. However, the lack of comprehensive genetic analyses on their adaptive and phenotypic traits has hindered the effective conservation and utilization of these resources. Using whole-genome resequencing, we systematically studied seven Tibetan sheep breeds, uncovering their genetic structure and diversity. Population analyses, including NJ and maximum likelihood trees, revealed clear genetic differentiation and migration patterns. Selective sweep analyses (Fst and θπ) identified hypoxia-related genes (DOCK8, IGF1R, JAK1, SLC47, TMTC2, and VPS13A) and wool color genes (TCF25, MITF, and MC1R). GWAS further detected candidate genes for body size traits (height, length, weight), enriched in cGMP-PKG, cAMP, and Hedgehog signaling pathways. Integrating GWAS and transcriptomics, we pinpointed key wool trait genes, including WNT16 (non-synonymous mutations), PRKCA, MAP3K8, MMP7, OVOL2 (intergenic SNPs), and COL7A1, KDM8, ZNF385D (intronic SNPs). Notably, HOX family transcription factors were found to critically regulate hair follicle development. These genetic markers offer promising targets for molecular breeding to enhance wool quality and adaptive traits. Our findings provide a genetic basis for understanding Tibetan sheep’s unique adaptations and production traits, supporting future breeding strategies and sustainable utilization of their genetic resources.

## Introduction

With the migration of human populations, particularly nomadic groups, domesticated sheep (*Ovis aries*) were first domesticated in the Fertile Crescent approximately 9,500 to 10,000 years ago (BCE, BP). Genetic analyses have revealed multiple distinct domestication lineages that have subsequently dispersed nearly worldwide (1). Tibetan sheep gradually disseminated from the northeastern region of the Tibetan Plateau to its central area as the Di-Qiang people expanded 3,100 years ago, and subsequently from the southwestern region to the central area by 1,300 years ago (2, 3). Tibetan sheep, inhabiting the relatively isolated Tibetan Plateau, have developed rich genetic resources and distinct local varieties due to natural geographical barriers and limited external species intrusion. Over time, these sheep have undergone both natural and artificial selection, resulting in significant phenotypic diversity, including variations in hair type, hair color, horn morphology, and tail structure, etc. The diverse phenotypes of Tibetan sheep, along with the extensive variation in economically important traits, offer researchers a wealth of genetic resources.

Previous research has identified approximately 17 local Tibetan sheep populations on the Qinghai-Tibet Plateau (4). As the exploration of germplasm resources continues, an increasing number of new germplasms have been discovered, including the Zashgar, Zeku, and Maduo sheep. These breeds, belonging to distinct groups within the Tibetan sheep population, have since evolved into independent breeds. Tibetan sheep breeds on the Qinghai-Tibet Plateau exhibit remarkable traits including drought tolerance, cold resistance, roughage adaptability, disease resilience, high-altitude acclimatization, foraging capability, robust physique, and strong genetic adaptability (5). Furthermore, Tibetan sheep hold significant agricultural, economic, cultural, and religious value in the Tibetan Plateau region of China, contributing substantially to the economic development of pastoral areas (6).

Artificial selection has significantly influenced the genetic diversity of sheep during domestication and production-oriented breeding, resulting in populations with distinctive characteristics and valuable genetic resources. The genome of Tibetan sheep offers a unique opportunity to identify traits associated with this selection. Under artificial intervention, the genetic variation affecting the fertility of consecutive multiple births in Tibetan sheep was investigated. It was discovered that PAPPA is a key gene responsible for stimulating the growth and development of ovarian follicles and enhancing steroid production, thereby improving reproductive success (7). A study examining genomic variation in 986 Tibetan sheep samples across their range identified strong selection signals in genes related to hypoxia and ultraviolet signaling pathways, including the HIF-1 pathway and the HBB and MITF genes. Additionally, strong selection was observed in genes associated with morphological traits, such as horn size and shape, particularly the RXFP2 gene. Furthermore, the study detected 5.23–5.79% gene introgression from argali (*Ovis ammon*) into Tibetan sheep (2). Genome-wide association analysis was conducted on 103 subtypes, including normal large horns, scurs, and polled, derived from the second generation (G2) of a Tibetan sheep polled core herd. Six SNPs located on chromosome 10 within the RXFP2 gene showed significant positive correlations with horn length, horn base circumference, and horn base interval (8). High-frequency structural variant genes, including EPAS1, PAPSS2, and PTPRD, represent significant sources of genetic variation in the gene expression of Tibetan sheep and play a crucial role in their adaptation to high-altitude environments (9). Positive selection genes related to meat production, coat color, wool traits, horn type or adaptability of different ecological types of Tibetan sheep have also been explored (10). During the successive generations of Tibetan sheep domestication, varying degrees of genetic imprints have been left on the genome, ultimately becoming fixed in certain domesticated breeds of Tibetan sheep. These valuable genetic resources delineate the genomic landscape of various ecological types of Tibetan sheep across their distribution range. However, each species may possess distinct candidate genes that are responsible for their unique traits, and there is a relative paucity of research on the selection markers for various economic traits across different ecological types.

In this study, we conducted whole-genome resequencing of Tibetan sheep populations from diverse ecological environments. Each sample possesses distinct traits, including hair length, high-altitude adaptation, body size, and coat color. Through the application of signal analysis methods for whole-genome scanning, we identified the specific genetic markers responsible for these traits in Tibetan sheep. This study enhances our understanding of the underlying genetic mechanisms and contributes to the broader field of fundamental biological research.

## Materials and methods

### Animal care

The study was conducted according to the guidelines of the Institutional Animal Care and Use Committee of Institute of Animal Science and Veterinary Medicine, Chinese Academy of Sciences (IACUC2021311).

### Animals and phenotypic measurement

**Figure 1:**
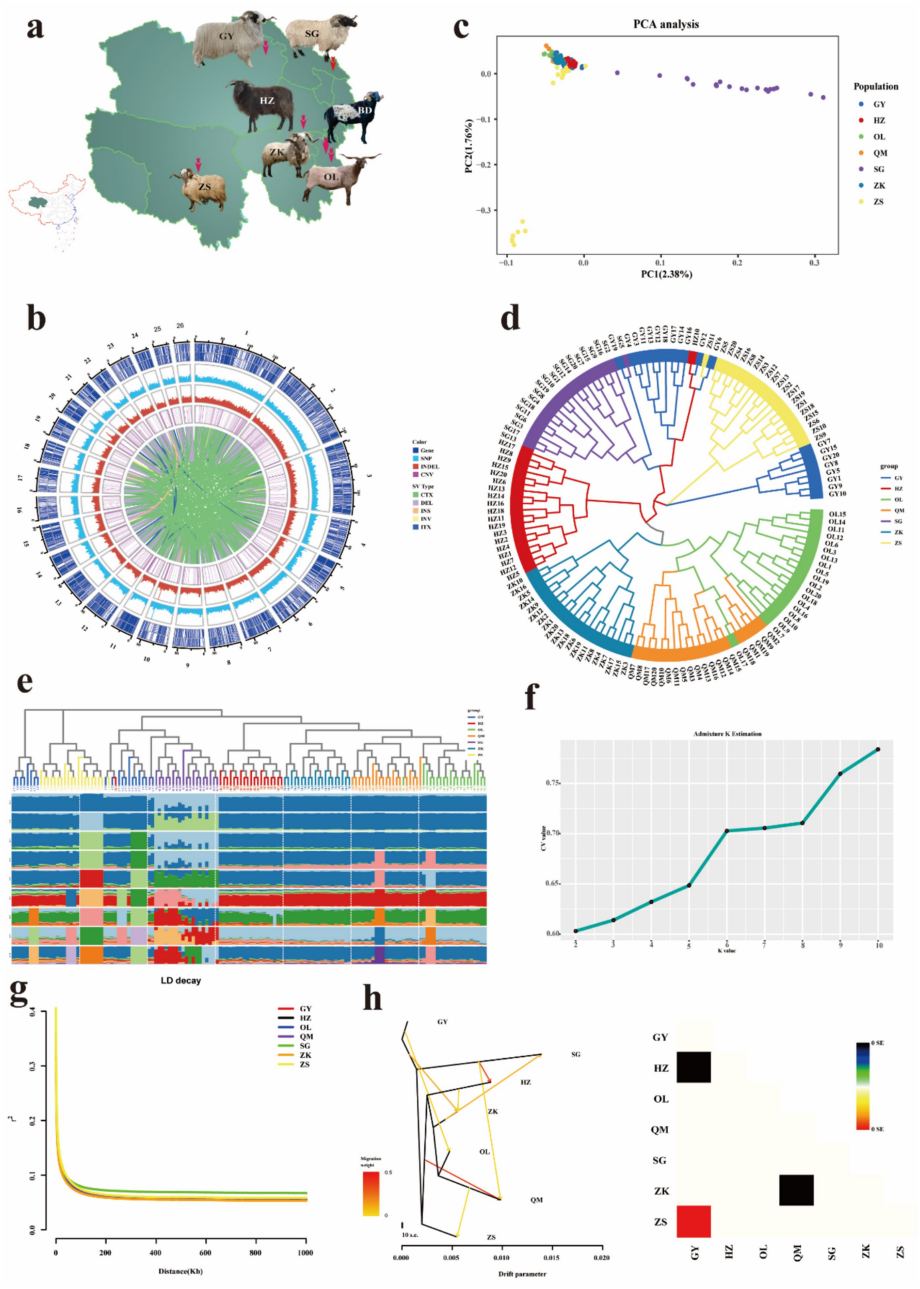
Geographic distribution and genetic diversity analyses. **(a)** Geographic distribution of seven Tibetan sheep breeds in Qinghai. The map was generated using Adobe Illustrator software. **(b)** SNPs and InDels density distribution circle. **(c)** Plots of principal components. **(d)** Neighbor-joining tree constructed from SNP data among six sheep populations. **(e)** Genetic structure analysis of samples using admixture, with changing ancestral populations from *K* = 2 to *K* = 10. **(f)** Distribution of cross-validation error values associated with varying *K* values. **(g)** The linkage disequilibrium (LD) decay analysis. **(h)** Gene flow diagram and residual fitting heat map.

Seven Tibetan sheep breeds exhibiting significant geographical and phenotypic variations were selected from Qinghai Province (plateau-type Tibetan sheep, valley-type Tibetan sheep, Black Tibetan sheep, Qumaari Speckled sheep, Zeku sheep, Oula sheep, and Zashgar sheep). A total of 140 blood samples were collected from unrelated adult ewes (*n* = 20 per breed). Each sample was meticulously documented with the species name, codes, geographical coordinates of the sampling site (longitude, latitude, and altitude), and phenotypic characteristics (see Figure 1a and Supplementary Table 1 for further details). All tissue samples were preserved in 95% ethanol and stored at −80°C for subsequent genomic analysis. The phenotypes of five traits were measured in 20 different breeds of Tibetan sheep, including three body size traits (body weight, body length, and body height) and two wool quality traits (wool fiber length and fiber fineness).

### Whole-genome resequencing

Total genomic DNA was extracted from the samples, and at least 3 μg of genomic DNA was used to construct paired-end libraries with a read length of 2 × 150 bp for paired-end sequencing. Simultaneously, we obtained the farmer’s consent to collect skin samples. We randomly selected three unrelated plateau Tibetan sheep and three unrelated Oula sheep, and collected 2 square centimeters of skin tissue from the shoulder region using sterile surgical blades. The samples were promptly immersed in liquid nitrogen (−80°C) for subsequent transcriptome sequencing and real-time quantitative polymerase chain reaction (RT-qPCR) analysis. These libraries were sequenced utilizing the Illumina NovaSeq 6000 platform located in Shanghai, China.

### Read processing and variant calling

ASTP Toolkit v0.18.0 was utilized for stringent quality control of the raw reads based on the following criteria: (1) removal of reads containing ≥ 10% unidentified nucleotides (N); (2) elimination of reads with > 50% bases having Phred quality scores ≤ 20; and (3) discarding reads that aligned to the barcode adapter. The Burrows–Wheeler Aligner (BWA) was employed to map the cleaned reads from each sample to the CAU O.aries_1.0 reference genome^1^ using the parameters “mem 4 -k 32 -M”. Here, -k specifies the minimum seed length, and -M marks shorter split alignment hits as secondary alignments (11). Variant calling was conducted across all samples using GATK’s Unified Genotyper (12). Subsequently, SNPs and InDels were filtered through GATK’s Variant Filtration tool, applying specific thresholds (-Window 4, -filter “QD <2.0||FS >60.0||MQ <40.0,” -G_filter “GQ <20”), and variants showing segregation distortion or indicative of sequencing errors were excluded (13). For the determination of the physical positions of each SNP, the ANNOVAR software (14) was applied for alignment and annotation of SNPs or InDels. Structural variations (SVs), encompassing translocations, inversions, and insertions, were identified using the breakdancer software (Max1.1.2.) (15). Copy number variants (CNVs) were classified using CNVnator (0.3.2) (16).

### Population genetic analyses

The SNP-only dataset was analyzed utilizing a maximum likelihood algorithm. A phylogenetic tree was constructed using PHYML 3.0 (17) and FastTree, based on the selected optimal nucleotide substitution model, generalized time reversible (GTR). Node support was assessed through 1,000 bootstrap replicates. Principal component analysis (PCA) was conducted using SNP data [excluding SNPs with a minor allele frequency (MAF) less than 0.05] via the GCTA software. Individuals were subsequently clustered into distinct subgroups based on the principal components derived from the analysis. The ADMIXTURE software^2^ was utilized to analyze the genetic structure of the population using SNP data. A mixed model was selected, with *K* ranging from 2 to 10 (assuming 2 to 10 ancestral populations). All other parameters were set to the software’s default values. The optimal *K* value, which is closest to the true number of ancestral populations, was determined based on the cross-validation (CV) error values.

### Selected sweep

Select regions with extremely low or high θ, where the π ratio is in the 5% left and right tails, and those with significantly high Fst values (i.e., the top 5% of Fst), as these are identified as regions that have undergone strong selective sweeps.

### Enrichment analysis of selected candidate genes

Use top GO/KEGG to conduct enrichment analysis. In this process, the gene list and gene count for each pathways are derived from the candidate region genes annotated. The *p*-value is then calculated using the hypergeometric distribution method, with a threshold of *p*-value <0.05 indicating significant enrichment. This approach identifies the pathways that are significantly enriched in the candidate region genes relative to the whole-genome background, thereby elucidating the primary biological functions of these genes.

### GWAS analysis

Based on inter-population SNPs and linkage disequilibrium, the EMMAX software was utilized to perform association analysis between molecular markers and trait phenotypes,^3^ identifying markers or candidate genes closely associated with the target traits. These findings were visualized using Manhattan plots and QQ plots.

### RNA extraction, library construction

Total RNA was extracted using the Trizol Reagent (Invitrogen Life Technologies). Subsequently, the concentration, quality, and integrity of the RNA were assessed using a NanoDrop spectrophotometer (Thermo Scientific). To isolate cDNA fragments of the desired 400–500 base pairs in length, the library fragments were purified utilizing the AMPure XP system (Beckman Coulter, Beverly, CA, United States). Products were purified using the AMPure XP system and quantified with the Agilent High Sensitivity DNA Assay on a Bioanalyzer 2100 (Agilent Technologies). Subsequently, the sequencing library was subjected to sequencing on the NovaSeq X Plus platform (Illumina).

### Library construction and sequencing

Fastp (18) (version 0.18.0) was used to filter adapters or low quality bases from raw reads. The reference genome and gene annotation files were retrieved from the genome database. The filtered reads were aligned to the reference genome using HISAT2 version 2.0.5.

### Differential expression analysis

We utilized HTSeq (version 0.9.1) to compare the read count values for each gene as a measure of its original expression, followed by normalization using FPKM. Differential gene expression was analyzed using DESeq (version 1.30.0) with the following screening criteria: an absolute log2 fold change greater than 1 and a significant *p*-value less than 0.05. Additionally, we employed the R language Pheatmap (version 1.0.8) package to conduct bi-directional clustering analysis of all differentially expressed genes across samples. The heatmap was generated based on the expression levels of the same gene in different samples and the expression patterns of different genes within the same sample, using the Euclidean method to calculate distances and the complete linkage method for clustering.

### Analysis integrating GWAS and transcriptomic data

The SNPs identified through GWAS were mapped to the corresponding genes in the expression dataset to evaluate their impact on gene expression. The significant genes obtained from GWAS were intersected with differentially expressed genes (DEGs), and KEGG/GO enrichment analysis was performed on the overlapping genes. Cluster analysis of the commonly differentially expressed genes was carried out using the Pheatmap package (version 1.0.8) in the R programming language.

### RT-qPCR analysis

Seven DEGs were selected and confirmed by RT-qPCR. The housekeeping gene actin served as an internal control for normalizing mRNA expression levels. Primers were designed using Oligo 6.0 software (Supplementary Table 30). Quantitative real-time PCR was performed in a 20 μL reaction volume containing 10 μL of 2 × Top Green EX-Taq Mix, 2 μL of cDNA, 7 μL of ddH_2_O, and 0.5 μL each of forward and reverse primers. The thermocycling conditions were as follows: initial denaturation at 94°C for 30 s; followed by 42 cycles of denaturation at 94°C for 5 s, annealing at 61°C for 35 s; a final extension at 97°C for 10 s, and a dissociation curve analysis stage consisting of 65°C for 60 s and 97°C for 1 s. Relative mRNA expression levels were determined using the 2^−ΔΔCT^ method. All experiments were conducted with six biological replicates.

## Results

### Sequencing, mapping and SNP/InDel detection

Whole-genome resequencing of 140 samples generated 36.4 billion paired-end raw reads with an insert size of 400 bp, and 35.98 billion high-quality reads, achieving an average depth of 14.44 × per sample and an average genome coverage of 99.33%. The GC content varied between 42.77 and 44.30%, the Q20 value was no less than 97%, the Q30 value was at least 93.69%, and the mapping rate against the reference genome surpassed 99.84% (Supplementary Table 2). Furthermore, a total of 46,681,283 SNP sites were identified for purine-pyrimidine transitions and transversions, with a Ts/Tv ratio of 1.86 (Supplementary Table 3). A total of 46.69 million SNPs were identified and utilized for subsequent analyses. The majority of these high-quality SNPs (64.35%) were found in intergenic regions, characterized by T/C and A/G transitions, while only 0.67% were located within exonic regions. The remaining SNPs were distributed as follows: 0.58% upstream, 0.56% downstream, and 33.82% within introns. In total, 314,431 SNPs were detected in exons, of which 46.63% were non-synonymous and 51.09% were synonymous, yielding a non-synonymous to synonymous ratio of 0.913 (Supplementary Table 4). In addition, a total of 1,880,322 insertions and 2,680,046 deletions were identified across seven indigenous sheep breeds (Supplementary Table 5).

The majority of insertions and deletions were situated in intergenic regions (63.71%). Additionally, exon InDels predominantly comprised in-frame deletions or insertions (56.62%), leading to alterations in the reading frame of protein-coding genes, which often exhibited a multiple of the triplet codon length (Supplementary Table 6). After quality control, the following copy number variants (CNVs) were detected: 24,803 in GY, 238,655 in HZ, 24,984 in OL, 24,960 in QM, 23,435 in SG, 22,029 in ZK, and 24,868 in ZS (Supplementary Table 7). A total of 337,491 reliable structural variants (SVs) were identified. Specifically, this included 3,469 insertions, 186,018 deletions, 15,204 inversions, 46,226 intrachromosomal translocations, and 86,574 interchromosomal translocations (Supplementary Table 8).

### Genetic diversity analysis and population genetic structure

The observed heterozygosity (Ho) of seven Tibetan sheep populations ranged from 0.358 to 0.372, while the expected heterozygosity (He) ranged from 0.349 to 0.358 (Supplementary Table 9). The observed heterozygosity was consistently higher than the expected heterozygosity, Fis values for ZS, SG, and GY were all less than 0. To gain a more comprehensive understanding of the distribution of chromosomal variations in Tibetan sheep, we established a 1 Mb sliding window and generated density maps for genes, SNPs, CNVs, and InDels within each window (Figure 1b). Notably, the genomic variation exhibits a high degree of homogeneity. The constructed weighted phylogenetic tree elucidates the relationships among seven Tibetan sheep breeds, and the resulting neighbor-joining (NJ) tree provides evidence of distinct separations between breeds, with each breed forming its own branch, although some overlap is observed in Tibetan sheep populations (Figure 1d). On the other hand, PCA cluster analysis offers further empirical support for the delineation of these groups, thereby demonstrating the robust consistency of the selected samples (Figure 1c). The mixed model was employed to assess the genetic structure of the population. At *K* = 2, the cross-validation error reached its minimum, resulting in the division of 140 germplasm resources into two distinct groups. When *K* = 3, SG tends to diverge in a different direction from the main group, while the remaining breeds are distributed across the other two clusters. When *K* = 7, the majority of the genetic information of GY, HZ, OL, QM, ZK, and ZS appears to stem from a shared ancestral population (red). When *K* = 10, the Tibetan sheep population exhibits extremely rich genetic diversity, which may be attributed to its highly complex population history, extensive geographical distribution, or diverse adaptive traits (Figures 1e,f). Linkage disequilibrium (LD, *r*^2^) decreases to half of its maximum value within less than 10 kb, with ZK populations exhibiting the fastest decline and SG populations showing the slowest decline. These findings indicate that SG species exhibit strong LD and are more prone to inbreeding, which may be a result of long-term artificial selection (Figure 1g). The maximum likelihood tree, based on the number of optimal migration events, provided evidence of gene flow between distinct Tibetan sheep populations (Figure 1h).

### Genome-wide study of selective sweeps for hypoxic adaptability

**Figure 2:**
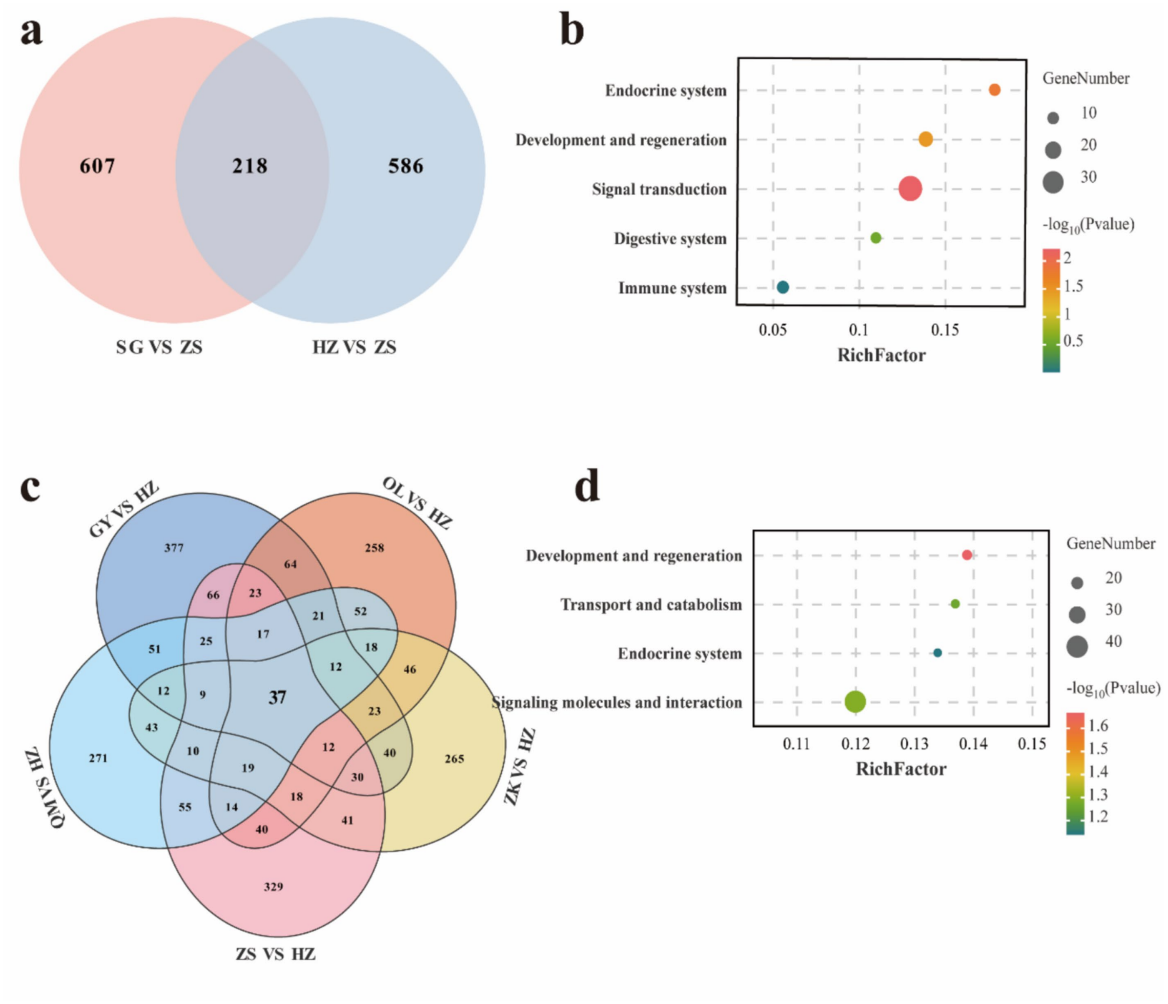
Venn diagrams of co-selected genes for hypoxic adaptability **(a)** and KEGG enrichment of the corresponding genes **(b)** among different comparisons. Venn diagrams of common selected genes for coat color **(c)** and KEGG enrichment of the corresponding genes **(d)** among different comparisons.

Through the analysis of genomic regions, we identified loci with high levels of Fst and nucleotide diversity ratio (θπ), which are associated with hypoxic adaptability. Specifically, by comparing varieties below 3,000 meters and those above 4,000 meters, we identified 2,900 and 2,618 candidate positively selected regions in the SG (Fst >0.082, θπ >0.496) (Supplementary Table 9) and HZ (Fst >0.044, θπ >0.365) (Supplementary Table 10) varieties, respectively. These two varieties exhibited 607 and 586 candidate genes, respectively, during variety-specific selection events, with a total of 218 co-selected genes (Figure 2a). These shared genes were selected through critical adaptive responses to hypoxic conditions in high-altitude environments, exhibiting higher levels of differentiation. For instance, several positively selected genes associated with hypoxic adaptation were identified in one or more of the studied populations, including DOCK8, IGF1R, JAK1, SLC4A7, TMTC2, and VPS13A. KEGG enrichment analysis revealed that these genes were predominantly enriched in signal transduction, development and regeneration, and endocrine system (Figure 2b and Supplementary Table 11).

### Genome-wide study of selective sweeps for coat color

Considering the physical properties of coat color, we employed a comprehensive comparative analysis, evaluating the OL, GY, HZ, ZS, and ZK varieties against the black-coated HZ variety. For OL (Fst >0.037, θπ <−0.305), GY (Fst >0.039, θπ >0.273), QM (Fst >0.039, θπ <−0.250), and ZS (Fst >0.044, θπ <−0.257) and ZK (Fst >0.039, θπ <−0.363), a total of 2,136, 2,625, 2,193, 2,376, and 2,034 selection regions were identified (Supplementary Tables 12–16), encompassing 37 co-selection genes (Figure 2c). Among the shared genes, including TCF25, MITF, and MC1R, which are associated with hair color, these genes are involved in transport and catabolism, regulation of the endocrine system, development and regeneration processes, as well as signaling molecules and interaction pathways (Figure 2d and Supplementary Table 17).

### Genome-wide association studies of body size and wool traits

**Figure 3:**
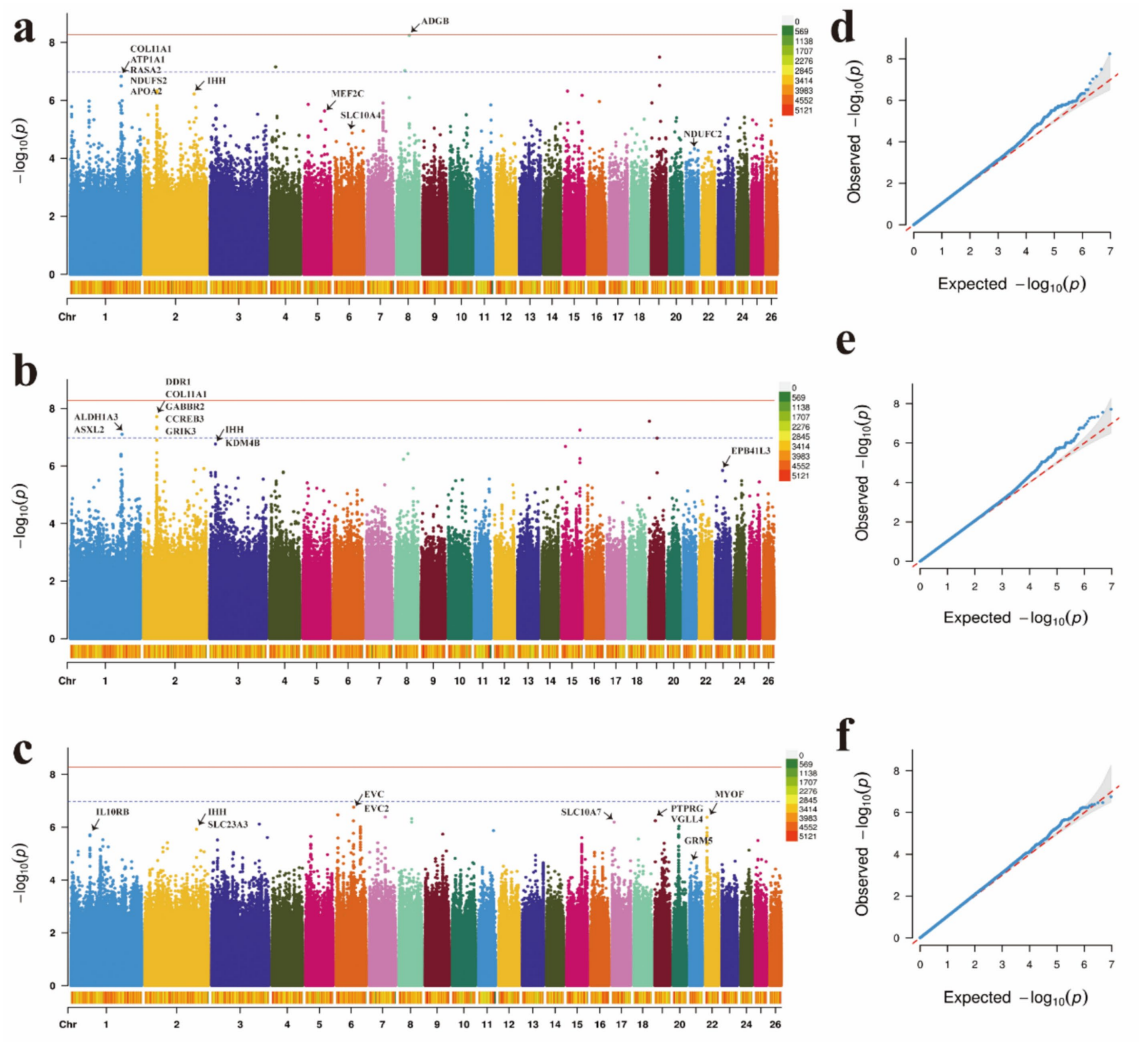
Manhattan plots and QQ plots of **(a)** body height, **(b)** body length, and **(c)** body weight traits. **(d)** QQ plots of the body height, **(e)** QQ plots of the body length, **(f)** QQ plots of the body weight.

GWAS serve as a crucial tool for elucidating the genetic underpinnings of traits, revealing the relationship between genetic variation and these traits. To account for known confounding factors, we incorporated gender and age as fixed effects into the mixed linear model using a general linear modeling approach. Based on the significance criterion of a *p*-value with a −log10 value exceeding 4, we identified several significant SNP loci associated with body height (BH), body length (BL), and body weight (BW) at the genomic level (Supplementary Tables 18–20). The *p*-value Manhattan plot revealed significant associations between SNPs and BH, BL, and BW. Through gene annotation, 30 significant loci related to body shape traits were identified. For the BH trait, five significant SNPs were located on OAR1, while one SNP was found on OAR2, OAR5, OAR21, and OAR6 (Figures 3a,d). For BL, five SNPs showed significant associations, with candidate regions identified on OAR1, OAR3, and OAR23. Notably, the five most significant SNPs were located on OAR2 (Figures 3b, e). For BW, 10 significant SNPs were identified, with the most prominent associations occurring in the EVC and EVC2 regions on OAR6 (Figures 3c,f). These SNPs demonstrated a strong positive correlation with body size traits (Table 1), exceeding the predefined threshold for significance.

**Table 1:** Significant SNPs associated with body traits.

Trait	Chr	Position (bp)	*p*-value	Nearest gene distance (bp)
BH	8	50,337,418	5.73 × 10^−9^	ADGB + 744
	1	203,095,849	1.49 × 10^−7^	APOA2 within
	1	203,095,849	1.49 × 10^−7^	ATP1A1 within
	1	203,095,849	1.49 × 10^−7^	Col11A1 within
	2	52,898,365	4.68 × 10^−7^	IHH
	5	17,204,283	1.38 × 10^−6^	MEF2C within
	21	34,264,023	4.70 × 10^−5^	NDUFC2 within
	1	203,095,849	1.49 × 10^−7^	NDUFS2 within
	1	203,095,849	1.49 × 10^−7^	RASA2 within
	6	115,451,273	1.13 × 10^−5^	SLC10A4 within
BL	1	206,870,312	7.89 × 10^−8^	ALDH1A3 within
	1	206,870,312	7.89 × 10^−8^	ASXL2 within
	2	52,780,121	1.93 × 10^−8^	Col11A1 within
	2	52,780,121	1.93 × 10^−8^	CREB3 within
	2	52,780,121	1.93 × 10^−8^	DDR1 within
	23	29,195,137	1.44 × 10^−6^	EPB41L3 within
	2	52,780,121	1.93 × 10^−8^	GABBR2 within
	2	52,780,121	1.93 × 10^−8^	GRIK3 within
	3	23,040,533	1.73 × 10^−7^	IHH within
	3	23,040,533	1.73 × 10^−7^	KDM4B within
BW	6	68,856,427	1.74 × 10^−7^	EVC
	6	68,856,427	1.74 × 10^−7^	EVC2
	21	6,487,822	2.24 × 10^−5^	GRM5
	2	204,617,524	1.19 × 10^−6^	IHH + 874
	1	75,251,362	1.93 × 10^−6^	IL10RB within
	22	5,907,806	4.22 × 10^−7^	MYOF within
	19	2,169,213	5.72 × 10^−7^	PTPRG within
	17	8,005,274	6.42 × 10^−7^	SLC10A7 within
	2	204,617,524	1.19 × 10^−6^	SLC23A3 + 874
	19	2,169,213	5.72 × 10^−7^	VGLL4 within

**Figure 4:**
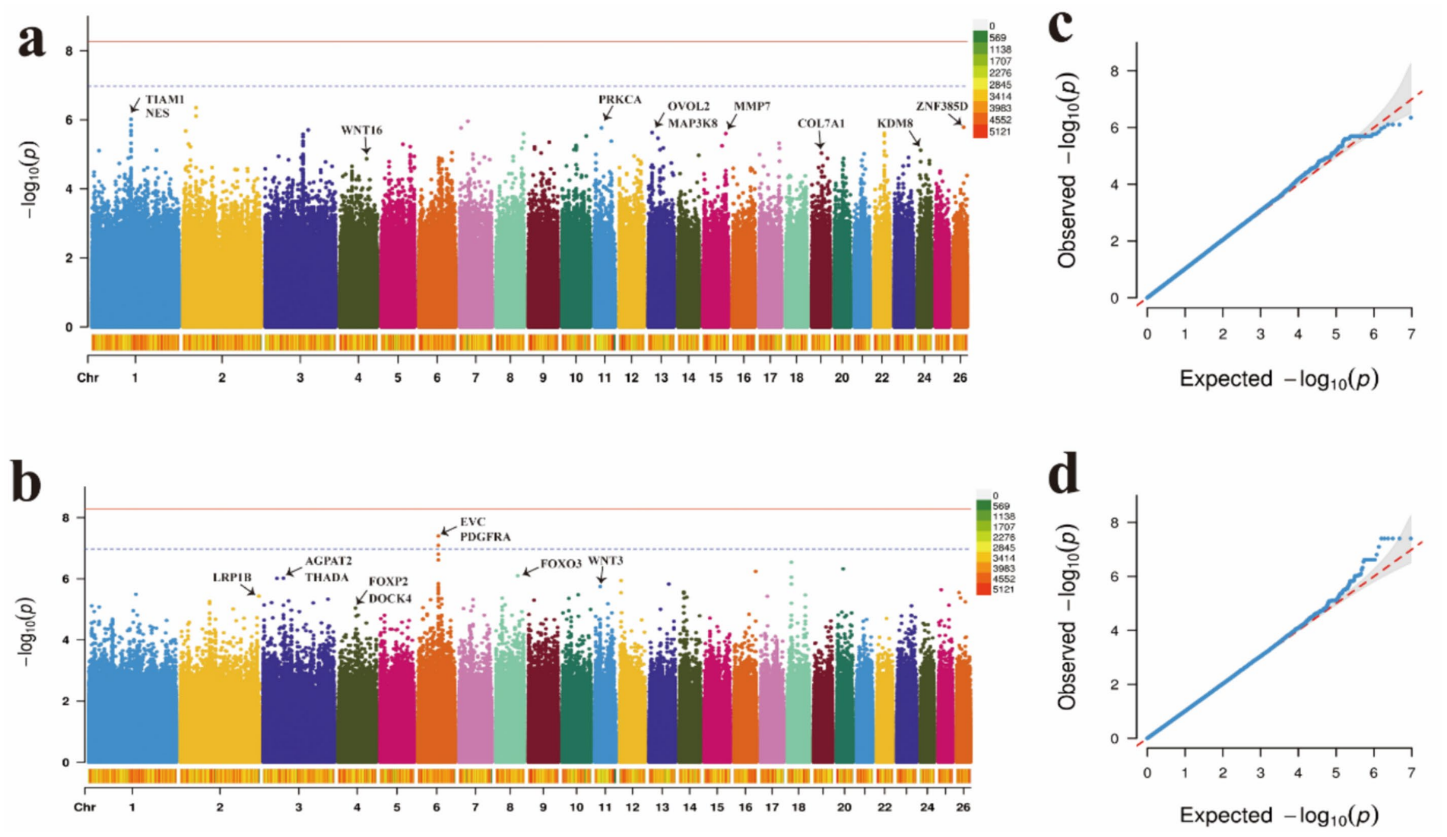
Manhattan plots and QQ plots of **(a)** wool fiber length, **(b)** wool fiber fineness traits, and **(c)** wool fiber length. **(d)** QQ plots of the wool fiber fineness.

Similarly, through GWAS analysis, several key genes associated with wool traits and their genetic variations were identified (Supplementary Tables 21, 22). Further annotation analysis revealed 19 significant loci linked to these traits. For wool fiber length (WL), the two most significant SNPs were located on OAR1, while one SNP was identified on each of OAR26, OAR11, OAR15, OAR24, OAR19, and OAR4, and two SNPs were found on OAR13 (Figures 4a,c). Regarding wool fiber fineness (WF), five SNPs were significantly associated with candidate regions on OAR8, OAR11, and OAR2, with the two most significant SNPs located on OAR6 (Figures 4b,d). We identified a set of significantly correlated SNP loci, with detailed information regarding these significant SNPs presented in Table 2.

**Table 2:** Significant SNPs associated with body traits.

Trait	Chr	Position (bp)	*p*-value	Nearest gene distance (bp)
WL	1	126,438,951	9.63 × 10^−7^	TIAM1
	1	126,438,951	9.63 × 10^−7^	NES
	26	33,042,355	1.63 × 10^−6^	ZNF385D
	11	23,512,651	1.71 × 10^−6^	PRKCA within
	13	12,598,138	2.34 × 10^−6^	OVOL2 + 153,175
	13	12,598,138	2.34 × 10^−6^	MAP3K8 + 153,175
	15	73,471,559	2.50 × 10^−6^	MMP7 within
	24	10,393,840	7.58 × 10^−6^	KDM8
	19	32,240,321	9.24 × 10^−6^	COL7A1
	4	87,216,849	1.35 × 10^−5^	WNT16
WF	6	64,919,046	3.97 × 10^−8^	EVC within
	6	64,919,046	3.97 × 10^−8^	PDGFAR within
	8	69,634,158	7.96 × 10^−7^	FOXO3 within
	3	64,541,866	9.53 × 10^−7^	AGPAT2 within
	3	64,541,866	9.53 × 10^−7^	THADA within
	11	17,517,094	1.78 × 10^−6^	WNT3
	2	250,582,498	3.66 × 10^−6^	LRP1B within
	4	55,140,180	9.06 × 10^−6^	FOXP2 within
	4	55,140,180	9.06 × 10^−6^	DOCK4 within

**Figure 5:**
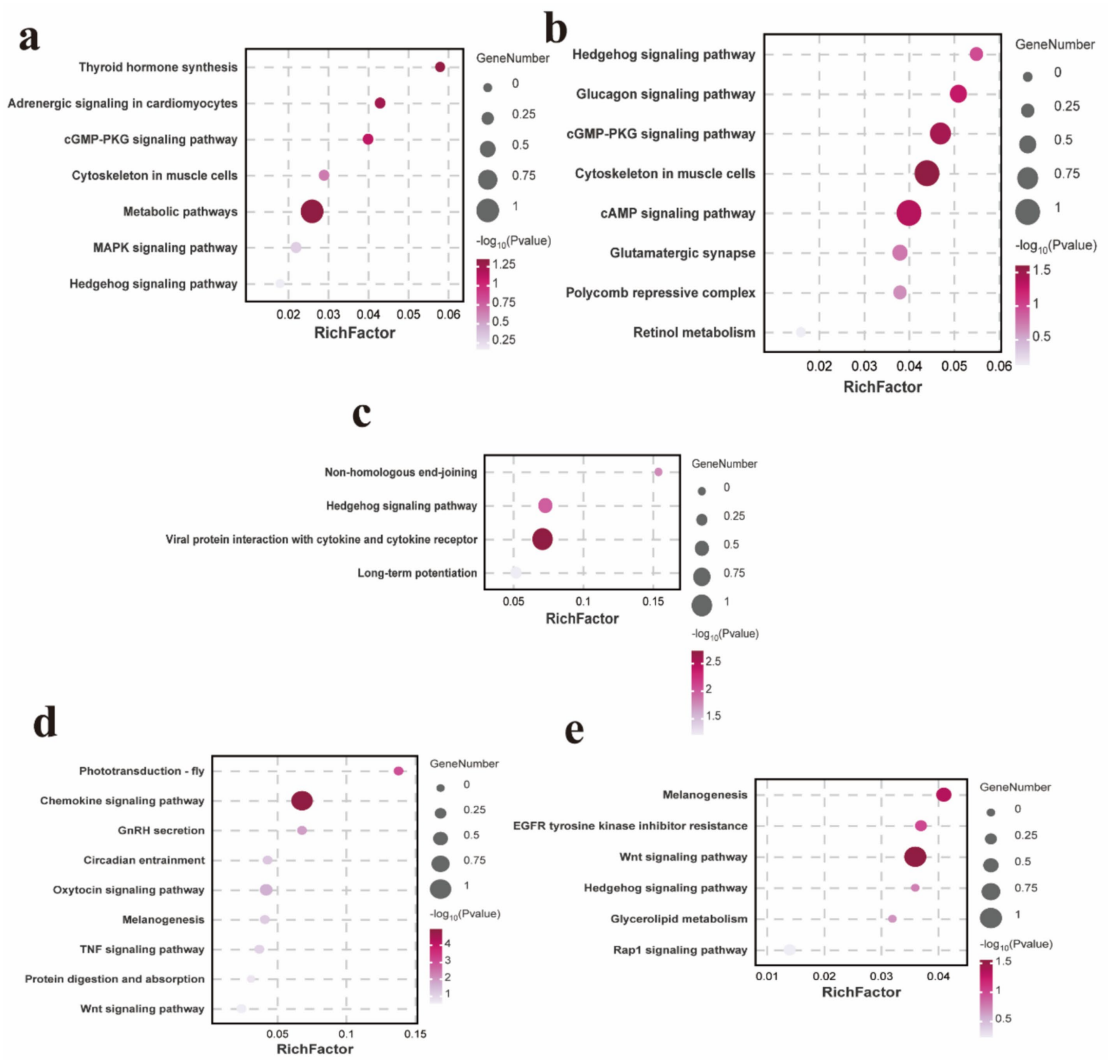
KEGG enrichment analysis pathway map of candidate genes associated with body height **(a)**. KEGG enrichment analysis pathway map of candidate genes associated with body length **(b)**. KEGG enrichment analysis pathway map of candidate genes associated with body weight **(c)**. KEGG enrichment analysis pathway map of candidate genes associated with wool fiber length **(d)**. KEGG enrichment analysis pathway map of candidate genes associated with wool fiber fineness **(e)**.

To comprehensively assess the characteristics of the candidate genes annotated by SNPs, we conducted enrichment analyses to identify the functional categories associated with these genes. In terms of body size traits, the enriched KEGG pathways are primarily associated with the cytoskeleton in muscle cells, cGMP-PKG signaling pathway, cAMP signaling pathway and hedgehog signaling pathway (Figures 5a–c and Supplementary Table 23). For wool fiber traits, the focus is mainly on the melanogenesis, TNF signaling pathway, Wnt signaling pathway, EGFR tyrosine kinase inhibitor resistance, hedgehog signaling pathway and Rap1 signaling pathway (Figures 5d,e and Supplementary Table 24).

### Analysis of RNA-seq data and functional annotation of DEGs associated with wool traits

**Figure 6:**
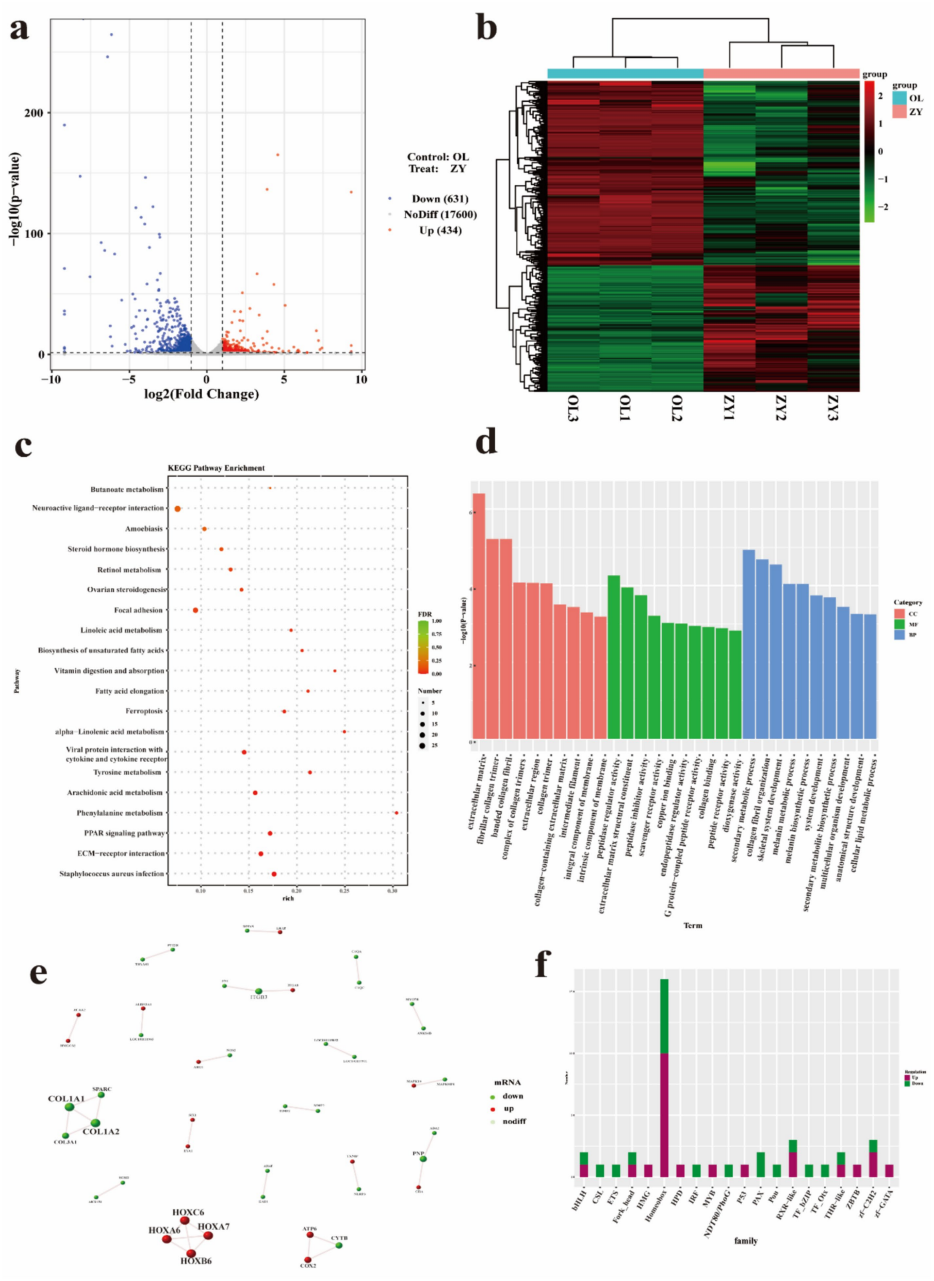
Skin transcriptome analysis. **(a)** Volcano plot of DEGs. **(b)** Clustered expression heatmaps of all mRNAs. **(c)** The top 20 KEGG pathways for DE mRNAs. **(d)** GO enrichment pathways for DE mRNAs. **(e)** PPI network of DEGs. **(f)** Histogram of differentially expressed transcription factors.

To elucidate the molecular mechanisms underlying the distinctive long hair traits of Tibetan sheep, we conducted transcriptomic analyses on skin samples from two Tibetan sheep breeds, ZY and OL, which exhibit significant differences in wool length. On average, 143,236,464 and 126,767,572 original reads were produced in the ZY and OL cDNA libraries, respectively. The GC contents of ZY and OL were 47.09 to 48.30% and 48.05 to 48.25%, respectively (Supplementary Table 25). Based on the significance threshold (|FC| >2 and *p*-value <0.05), a total of 1,065 DEGs were identified, including 434 up-regulated and 631 down-regulated genes (Figure 6a). The hierarchical clustering showed that there were significant differences in the expression profiles of DEGs between the treat group (GY) and the control group (OL) (Figure 6b). GO analysis revealed significant enrichment in several biological processes, including fibrillar collagen trimer, melanin metabolic process, peptidase regulator activity, and cellular lipid metabolism process (Figure 6c and Supplementary Table 26). A total of 39 significant pathways were identified through KEGG pathway analysis. The top 20 enriched pathways are presented in Figure 6d, indicating that DEGs (73 up-regulated and 155 down-regulated) were predominantly enriched in ECM-receptor interaction, PPAR signaling pathway, focal adhesion, fatty acid elongation and biosynthesis of unsaturated fatty acids these pathways (Supplementary Table 27). Based on the threshold for DEGs analysis, we identified and screened protein pairs with a node score greater than 0.95 in the STRING database to construct the PPI network (Figure 6e). According to the interaction results, there are four up-regulated gene modules (HOXC6, HOXA6, HOXA7, and HOXB6) and four down-regulated gene modules (COL1A1, COL1A2, COL3A1, and SPARC). Additionally, some modules contain both up-regulated genes (such as ATP6, COX2) and down-regulated genes (such as CYTB). Notably, the HOX homeobox genes, which encodes transcription factors (TFs), exhibited a significantly high degree of connectivity within this network. To further investigate the distribution characteristics of differentially expressed TFs, we conducted a statistical analysis of the number of differentially expressed TFs in each family within the control group. The results indicate that the homeobox family exhibits the most significant differential expression, with 10 upregulated TFs identified, including HOXC6, HOXC8, HOXA6, HOXA10, HOXC9, HOXA1, HOXB6, and HOXC10, as well as six downregulated factors (Figure 6f and Supplementary Table 28). Notably, the Hox subfamily demonstrates the most extensive distribution pattern within this family. This finding highlights the critical role of the HOX homeobox family, particularly its Hox subfamily, in gene regulatory networks.

### Identification of functional mutations in DEGs supported by whole-genome resequencing data

**Figure 7:**
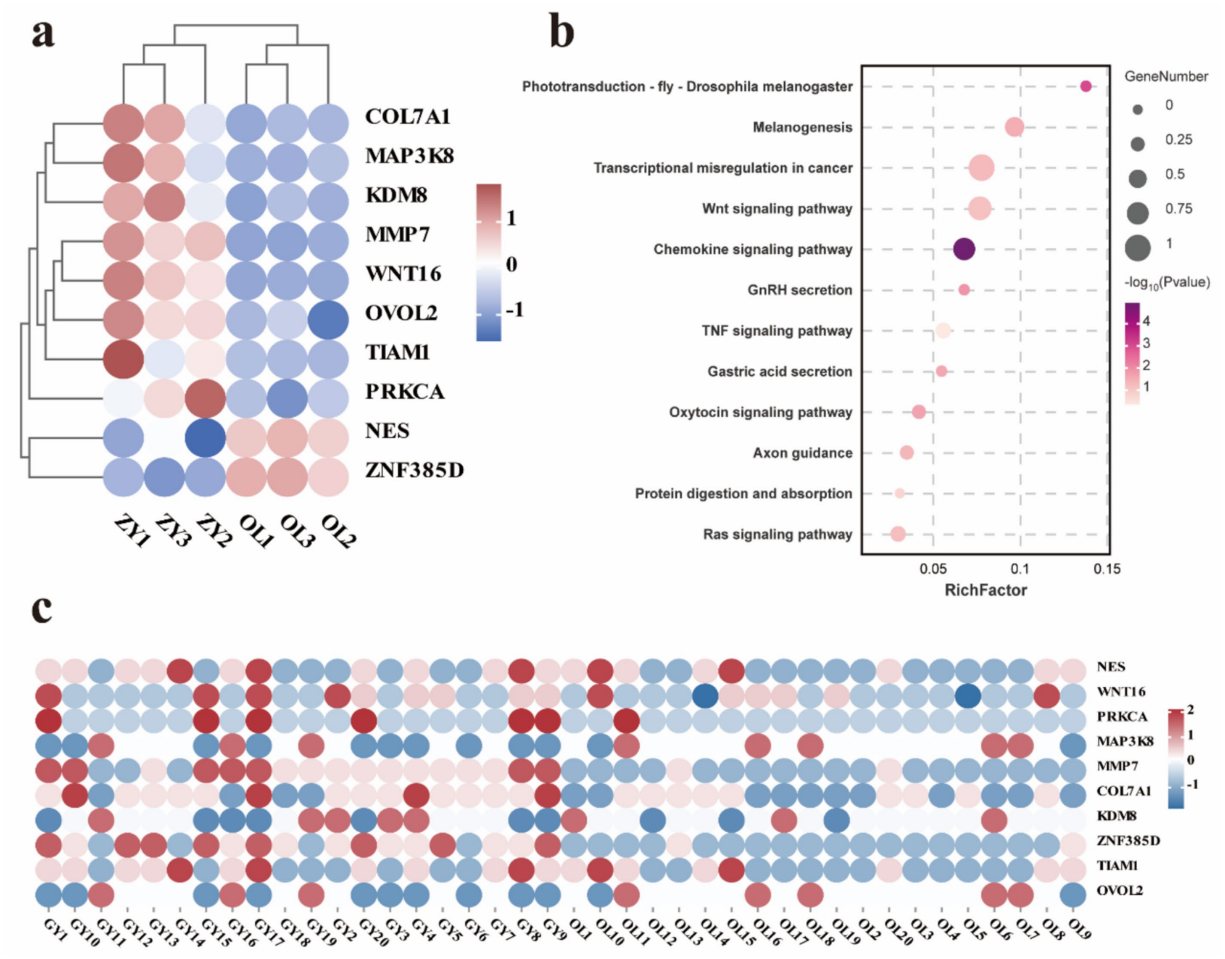
Analysis of key gene expression and variation. **(a)** Clustered expression heatmap of 10 key genes. **(b)** KEGG enrichment analysis of key expression genes. **(c)** Heatmap of variation of 10 key expressed genes.

To further evaluate the expression levels of candidate genes identified through GWAS, we examined the intersection between these candidate genes and DEGs associated with two distinct wool traits. SNPs were identified in critical regions of 10 key overlapping DEGs. For instance, a non-synonymous SNP in the coding region of WNT16 was predicted to alter the protein sequence, whereas synonymous mutations were observed in the coding regions of NES and TIAM1. SNPs were also detected in intergenic regions of PRKCA, MAP3K8, MMP7, and OVOL2, as well as within introns of COL7A1, KDM8, and ZNF385D (Table 3). The expression levels of 10 overlapping DEGs exhibited differential expression and distinct clustering patterns, with 8 genes up-regulated and 2 genes down-regulated (Figure 7a). Subsequently, KEGG enrichment analysis was conducted on these overlapping genes to elucidate their biological functions. The results indicate that these genes are involved in multiple metabolic pathways, including the Wnt signaling pathway, Ras signaling pathway, protein digestion and absorption, TNF signaling pathway, melanogenesis and chemokine signaling pathway (Figure 7b and Supplementary Table 29). Heat maps were utilized to visualize the genotypes of overlapping genes, and the results demonstrated that the polymorphism of the GY genotype was significantly higher compared to that of the OL genotype (Figure 7c).

**Table 3:** Shared genes exhibiting similar expression patterns and functional annotations.

Gene	Chromosome	SNV region	Expression	Description
WNT16	4	Exonic	Up	Protein Wnt-16
MMP7	15	Intergenic	Up	Matrilysin precursor
ZNF385D	26	Intronic	Down	Zinc finger protein 385D isoform X1
NES	1	Exonic	Down	Nestin isoform X1
COL7A1	19	Intronic	Up	Collagen alpha-1 (VII) chain
MAP3K8	13	Intergenic	Up	Mitogen-activated protein kinase kinase kinase 8
KDM8	24	intronic	Up	Bifunctional peptidase and arginyl-hydroxylase JMJD5
OVOL2	13	Intergenic	Up	Transcription factor Ovo-like 2
TIAM1	1	Exonic	Up	Low quality protein: T-lymphoma invasion and metastasis-inducing protein 1
PRKCA	11	Intergenic	Up	Protein kinase C alpha type isoform

### RT-qPCR validation of DEGs

**Figure 8:**
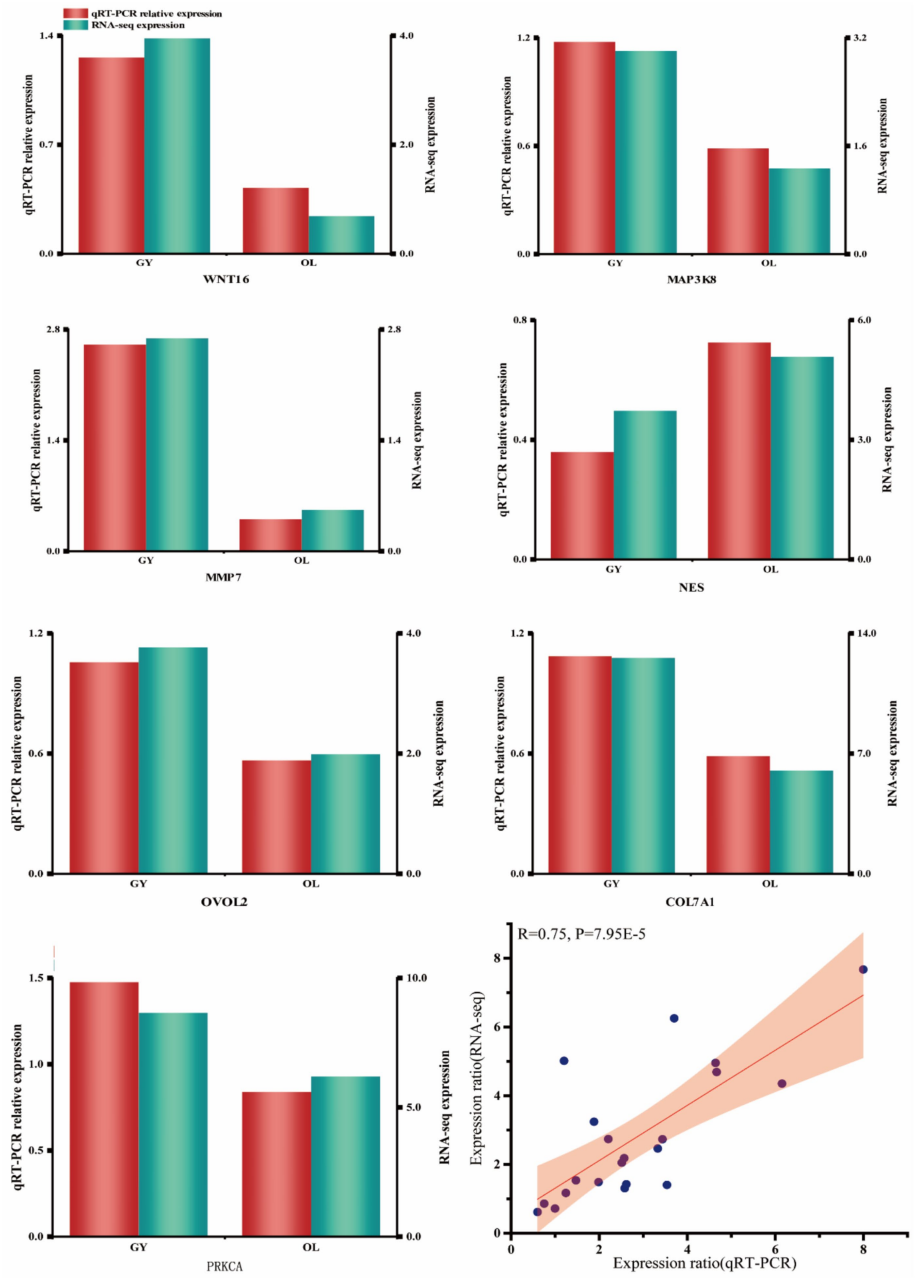
RT-qPCR validation of DEGs and correlation scatter plot of DEGs.

To validate the accuracy of the DEGs assay at the transcriptome level, we selected seven genes and evaluated their expression levels using six replicates for each sample. The results demonstrate a high degree of consistency between the two methods at the expression level, with a Pearson correlation coefficient of 0.75 (Figure 8). This indicates that the RNA-seq data exhibit strong reliability. The selected candidate genes exhibited strong concordance at both the transcriptional and actual expression levels, further substantiating their potential significance in subsequent functional analyses.

## Discussion

The genetic diversity of sheep constitutes the foundation of their evolutionary success. This diversity arises from both natural selection in response to diverse environmental pressures and artificial selection driven by human breeding programs designed to enhance economically valuable traits. There is compelling evidence indicating accelerated changes in specific genomic regions under artificial selection (19). A lower level of genomic diversity in domestic breeds compared to their wild ancestors, suggesting that a significant amount of genetic variation has been lost during and after the domestication process. While the genomic diversity of local breeds has largely been retained in improved varieties, contemporary breeding practices that focus on a limited range of commercial varieties have led to genetic homogenization. This increases the risk of adaptive allelic loss in native populations (20). Despite significant advancements in genomics, systematic studies comparing genetic divergence among geographically distinct sheep populations are still limited, especially for local breeds that possess unique ecological adaptations. In this study, we performed a comprehensive analysis of seven indigenous Chinese sheep breeds, including several Tibetan sheep ecotypes, to elucidate the genetic basis underlying their divergent traits such as hypoxic adaptation, growth performance, and wool characteristics, and to investigate their population structure. Our results revealed significant genetic differentiation among Tibetan sheep subpopulations, driven by both natural selection and historical pastoral management practices. Notably, genome-wide comparisons of local breeds have been limited, and our study addresses this gap by providing high-resolution insights into the genetic variation within these understudied populations. These findings underscore the indispensable role of the distinctive phenotypic traits of local sheep breeds in preserving the genetic diversity of sheep within our country. Additionally, the molecular markers identified through whole-genome resequencing that are significantly associated with key economic traits offer a robust foundation for developing a marker-assisted selection system. The seven native sheep breeds examined in this study have developed distinct adaptive phenotypic differentiation across varying elevations on the Tibetan Plateau. Among these, GY, ZK, HZ, and ZS, as typical high-altitude adapted breeds (≥3,000 m), exhibit longer wool fiber lengths and higher hair follicle densities. These traits likely represent a low-temperature adaptive strategy achieved through positive selection of genes associated with hair follicle development (21). In contrast, QM and OL display a “short coarse hair” phenotype, which, while reducing textile suitability, is significantly positively correlated with muscle growth efficiency, reflecting the selection pressure driven by the economic needs of herders (22, 23). Domestication, adaptation, and artificial selection have led to a diverse array of coat colors, which are the most distinctive characteristics among different breeds (24). The ancestral coat color of sheep was predominantly brown; however, domesticated breeds now exhibit a wide variety of colors and patterns (25). Notably, the QM breed primarily displays black-brown and yellow-brown coats, often with white spotting on the back, sides, and hips. Some studies suggest that this coloration may be influenced by upstream regions of the MITF gene and strong linkage disequilibrium with other loci (26), providing a novel model for studying the formation of body surface patterns in mammals.

Hypoxic adaptation in plateau animals involves a complex, multi-layered molecular regulatory network wherein key genes enhance oxygen utilization efficiency through synergistic interactions. IGF1R is a tyrosine kinase receptor located on the cell surface. Its binding to its ligands, IGF1 or IGF2, activates downstream PI3K/Akt and RAS/MAPK signaling pathways, thereby promoting cell proliferation, differentiation, migration, and survival while inhibiting apoptosis (27). Hypoxia can induce alterations in the IGF system, potentially due to diminished anabolic effects of IGFs. Additionally, increased expression of IGF1R may reflect a tissue-protective mechanism that compensates for changes in the IGF system, such as reduced serum levels of IGF-I and -II and elevated IGFBP-1 (28). Decreased expression of SLC4A7 under hypoxic conditions may compromise critical intracellular pH regulatory functions that cells normally maintain through substantial resource allocation. This adverse effect is particularly pronounced in the mammalian central nervous system, where hypoxia induces a spectrum of physiological responses that vary depending on the developmental stage (29). This upregulation suggests that enhanced fluid reabsorption may serve to prevent edema and inhibit T cell proliferation, thereby mitigating acute lung inflammation and facilitating adaptation to hypoxic conditions (30). DOCK8 functions as a negative regulator of HIF2α nuclear translocation in CD4^+^ T cells (31). VPS13A, JAK1, TMTC2, and other genes are implicated in multi-dimensional biological regulation, collectively mediating the complex, multi-level response mechanisms involved in hypoxia adaptation (32–34). The stable genetic adaptability of Tibetan sheep facilitates dynamic multilevel adaptive variations at the genomic level, thereby enhancing the species’ systemic ecological response efficiency to extreme high-altitude hypoxia stress.

The high diversity of domestic animal coat color results from a combination of artificial selection and adaptive evolution. This diversity largely reflects variations in human preferences or the fixation of certain colors associated with desirable domestication traits such as docility, reproductive rate, and growth rate. In the local Tibetan sheep population, coat colors include black, white, brown, and variegated phenotypes, with white being the most common. MITF serves as a critical regulator in the development, proliferation, and survival of melanocytes. Mutations in MITF can result in metabolic dysfunction of pigment cells, thereby impacting fur pigmentation (35). A synonymous MITF g.1548 C/T mutation was identified, and the frequency of the C allele was strongly associated with the pure white coat in Tibetan sheep, indicating that the C allele is likely the dominant allele for white coat color (36). Loss-of-function mutations in the MC1R gene result in a shift towards phaeomelanin synthesis, while SNPs at position 901C/T within the MC1R coding region are associated with white coat color (37). In mouse embryos, TCF25 is robustly expressed in the dorsal root ganglion. Based on this observation, we hypothesize that the regional differential expression of TCF25 may influence subsequent melanocyte migration, potentially leading to the formation of dark streaks along the midline of the back in goats (38).

Body size traits, including BH, BL, and BW, are critical indicators of production performance in livestock. Their genetic architecture is shaped by both natural selection (e.g., optimization of the body surface area-to-volume ratio for cold adaptation) and artificial selection (targeted breeding for economically important traits such as meat yield and feed conversion ratio). Variance estimation for sheep body size traits revealed heritability (*h*^2^) values of 0.22 ± 0.08 for BW, 0.11 ± 0.06 for BL, and 0.17 ± 0.06 for BH, with significant genetic correlations among these traits. The observed positive genetic and phenotypic correlations indicate that selection for body size traits can improve both genetic and phenotypic body size, making it a key target for modern molecular breeding (39). As a crucial approach in modern quantitative genetics research, GWA studies have showcased substantial advantages in the genetic analysis of body shape traits in sheep. In this study, by employing the mixed linear model analysis method, we systematically identified genetic variants significantly associated with body height, body length, and body weight in Tibetan sheep. These variants may represent key genetic factors influencing the development of body shape traits in Tibetan sheep. RASA2 is a compelling candidate gene for regulating height, as copy number variants (CNVs) and loss-of-function mutations in RASA2 have been associated with short stature (40). NDUFS2 is considered a functional candidate gene influencing meat quality, primarily governing phenotypes such as fat metabolism and muscle development, and playing a crucial role in energy metabolism synthesis (41). IHH, as a critical signal for the local growth of endochondral bone, regulates bone development through multiple parallel pathways. Disruption of IHH signaling leads to a progressive reduction in embryonic bone size (42). Inactivation mutations in Evc or Evc2 within the perichondrium result in markedly elevated FGF signaling, leading to severe dwarfism characteristic of Evc syndrome (43). COL11A1 plays an essential role in bone morphogenesis, and variations in this gene are linked to human height (44). ALDH1A3, ATP1A1, MEF2C, NDUFC2, EPB41L3, IL10RB, PTPRG, and GRM5 are likely to play a significant role in growth traits (45–52). The effects of the aforementioned diverse genes highlight the intricate genetic regulatory mechanisms underlying body size, a quintessential quantitative trait. The development of body size is a dynamic and multi-tiered regulatory process. Future studies can achieve a more comprehensive understanding of the genetic basis of body size by constructing gene regulatory networks and identifying functional modules.

In an integrated analysis of GWAS and transcriptome data, we combined genomic variation with gene expression profiles to identify a set of genes whose expression levels are significantly influenced by genetic variation. These candidate genes may contribute to phenotypic variation through the regulation of key biological pathways or molecular mechanisms. Long wool is a crucial genetic resource in Qinghai Tibetan sheep, characterized by its high content of medullated fibers. Consequently, Tibetan wool is extensively utilized in carpet manufacturing and renowned for its superior quality (53, 54). CircRNAs may play a crucial role in the development of hair follicles and the growth of cashmere by forming a balanced regulatory relationship with their host gene, TIAM1 (55). The long non-coding RNA (lncRNA) MSTRG.20890.1, transcribed from the intronic region of the ZNF385D gene, inhibits the proliferation and migration of dermal fibroblasts by competitively binding to chi-miR-24-3p with ADAMTS3. Consequently, this interaction leads to the inhibition of dermal papillary structure formation and secondary hair follicle morphogenesis (56). OVOL1-OVOL2 axis serves as a positive regulator in normal hair development and differentiation, facilitating the proliferation and differentiation of hair follicle cells. Therefore, OVOL1 and OVOL2 may represent potential therapeutic targets for the treatment of hair loss (57). Nestin (NES)-containing cells constitute the predominant cell population in the hair follicle throughout each follicle cycle, and nestin-expressing cells serve as stem cells for the entire hair follicle (58). The down-regulated expression of MAP3K8 inhibits the proliferation and melanin synthesis in sheep melanocytes (59). PKC plays a pivotal role in cellular signal transduction, modulating the proliferation and differentiation of hair follicle cells (60). It is noteworthy that in our study, the HOX homeobox genes, which encode TFs, exhibited exceptionally high connectivity within the protein–protein interaction (PPI) network and demonstrated the most significant differential expression. Previous research has found that HOX family TFs play a pivotal role in regulating cell differentiation, function, proliferation, embryonic development, and tissue homeostasis by modulating the promoter regions of multiple target genes (61). Specifically, in hair follicle biology, HOX homeobox genes are crucial for establishing the topological specificity of hair follicles and are integral to their development, cycle regulation, and regeneration (62–64). These genes may significantly influence hair follicle formation and function through the regulation of hair follicle stem cell activity, interaction with signaling pathways, and region-specific expression patterns. These findings underscore the important role of HOX genes in hair follicle biology and provide valuable insights for further research into the mechanisms of hair follicle development and regeneration. These results established a critical foundation for the subsequent validation studies and provided novel insights into the genetic analysis of sheep wool traits. Moving forward, we intend to conduct in-depth investigations of the identified candidate genes, encompassing gene function elucidation, regulatory network construction, and both *in vitro* and *in vivo* functional validations. This will further uncover the causal mutations influencing sheep wool traits and their associated molecular regulatory pathways, thereby offering alternative molecular targets and a robust theoretical basis for sheep breeding.

## Conclusion

Our experimental strategy is based on selective clearance analysis of whole genome resequencing and integrated analysis of GWAS and transcriptome data to identify key genomic regions and genes under selection in the Tibetan sheep genome. By combining genomic variation and gene expression profiles, we have successfully pinpointed candidate genes associated with wool traits and their regulatory pathways. These findings not only elucidate the genetic mechanisms driving the unique adaptability of Tibetan sheep to extreme environments but also offer novel insights into the molecular basis of economically important traits. Furthermore, our results provide a robust theoretical foundation for the conservation and utilization of sheep genetic resources, significantly advancing molecular breeding and genetic improvement efforts for Tibetan sheep.

## Data Availability

The raw reads produced in this study were deposited in the NCBI SRA with the accession number SRA SUB15389572 under Bio-project PRJNA1280483. Additional data can be found in Supplementary files.

## Footnotes

^1^ https://www.ncbi.nlm.nih.gov/datasets/genome/GCA_017524585.1/

^2^ http://dalexander.github.io/admixture

^3^ http://genetics.cs.ucla.edu/emmax/index.html
